# The potential habitat of *Angelica dahurica* in China under climate change scenario predicted by Maxent model

**DOI:** 10.3389/fpls.2024.1388099

**Published:** 2024-07-29

**Authors:** Fen-Guo Zhang, Furong Liang, Kefan Wu, Liyuan Xie, Guanghua Zhao, Yongji Wang

**Affiliations:** College of Life Science, Shanxi Engineering Research Center of Microbial Application Technologies, Shanxi Normal University, Taiyuan, Shanxi, China

**Keywords:** *Angelica dahurica*, MaxEnt model, climate factors, habitat prediction, climate change

## Abstract

Since the 20th century, global climate has been recognized as the most important environmental factor affecting the distribution of plants. *Angelica dahurica* (*A. dahurica*) has been in great demand as a medicinal herb and flavoring, but the lack of seed sources has hindered its development. In this study, we utilized the Maxent model combined with Geographic Information System (GIS) to predict the potential habitat of *A. dahurica* in China based on its geographical distribution and 22 environmental factors. This prediction will serve as a valuable reference for the utilization and conservation of *A. dahurica* resources.The results indicated that: (1) the Maxent model exhibited high accuracy in predicting the potential habitat area of *A. dahurica*, with a mean value of the area under the ROC curve (AUC) at 0.879 and a TSS value above 0.6; (2) The five environmental variables with significant effects were bio6 (Min temperature of the coldest month), bio12 (Annual Precipitation), bio17 (Precipitation of Driest Quarter), elevation, and slope, contributing to a cumulative total of 89.6%. Suitable habitats for *A. dahurica* were identified in provinces such as Yunnan, Guizhou, Guangxi, Sichuan, Hunan, and others. The total area of suitable habitat was projected to increase, with expansion primarily in middle and high latitudes, while areas of decrease were concentrated in lower latitudes. Under future climate change scenarios, the centers of mass of suitable areas for *A. dahurica* were predicted to shift towards higher latitudes in the 2050s and 2090s, particularly towards the North China Plain and Northeast Plain. Overall, it holds great significance to utilize the Maxent model to predict the development and utilization of *A. dahurica* germplasm resources in the context of climate change.

## 1 Introduction

Historical observations show that the global average temperature of land and sea increased by 0.78° from 1850 to 2012 (Xue et al., 2023; Shen and Wang, 2013). Since the 20th century, the global climate has shown a major trend of warming, and climate is considered to be the most important environmental factor affecting plant distribution. In the future, without significant reductions in greenhouse gas emissions, global surface temperature will increase by at least 2.1° by 2100 (Ma et al., 2022). Climate change is a serious problem at the global level, affecting entire organisms, including plants. Atmospheric concentrations of carbon dioxide and average temperatures have been rising over the past few decades, and this trend is expected to become more severe in the near future. In addition, environmental stresses such as drought, salinity, ultraviolet radiation, and exposure to heavy metals and toxic elements pose a threat to ecosystems and agriculture. Climate and environmental changes negatively affect plant growth, biomass and yields, and also increase plant susceptibility to pests and diseases (Bokor et al., 2021). Anthropogenic climate change leads to declines in ocean productivity, changes in food web dynamics and fewer habitat-forming species, resulting in changes in species diversity and ranges (Priya et al., 2023). The biodiversity of species is a fundamental component of nature and an important asset shared by all humankind. It not only has great economic value and provides a source of food for mankind; at the same time, species diversity is a valuable repository of genetic resources and provides a source of genes needed by mankind. In the context of climate change, it is therefore necessary to predict the impact of climate change on the potential geographic distribution patterns of plants. In the context of climate change, the potential distribution areas of plants will be changed accordingly, so it is necessary to predict the impact of climate change on the potential geographic distribution pattern of plants.

Species Distribution Models (SDMs) are mathematical models that predict the distribution of suitable habitats for a species by analyzing its distribution data and associated environmental factors. These models assess the survival needs of the species by considering the environmental information gathered from the species' distribution points, and then projecting this data onto a selected study area (Juan and Ni, 2006; Anderson, 2013; Guo et al., 2020; OuYang et al., 2022). As an important research method in biogeography and macroecology, the species distribution model plays an important role in the field of life sciences and environmental sciences. Among the many species distribution models, the Maximum Entropy model (MaxEnt) stands out due to its advantages of lower data requirements, higher prediction accuracy, and ease of use (Fu et al., 2016; Ma and Li, 2023). Species distribution models began with the BioCliM model, followed by generalized linear, additive, and MaxEnt models (Fu et al., 2016). MaxEnt model is a quantitative analysis tool with more applications in the prediction (Ma et al., 2010) of species distribution, species adaptive area, etc (Elith et al., 2006). It is used to predict the probability distribution of the target by calculating the probability distribution of maximum entropy, which is currently used to predict the distribution of the more ideal model. With the development of conservation biology, ecology and other disciplines, the MaxEnt model has been applied more to different species, different periods, and different needs of the situation (Ma et al., 2010; Zhang and Yang, 2018).

*Angelica dahurica* (*A. dahurica*), once a symbol of Chinese civilization, is a commonly used Chinese medicine with a long history and a wide range of applications. It was first published in Shennong Ben Cao Jing (Classic of the Divine Husbandman's Materia Medica), classified as a middle grade, and is also known as Sichuan *A. dahurica* and Walking Horse Celery, among other names (Wang et al., 2017; Zhang and Yang, 2018; Zou et al., 2023). As a member of the umbelliferae genus, *A. dahurica* is incredibly adaptable and thrives in warm and humid climates. It prefers to be planted in deep, loose, and fertile sandy loam soil (Wang et al., 2017; Ji et al., 2020). This herb is distributed in the northeast of China's mainland, North China, and other regions, typically growing at altitudes of 200 to 1500 meters above sea level. Depending on the region of origin, *A. dahurica* can be categorized as *A. dahurica* Chuan, *A. dahurica* Hang, *A. dahurica* Yu, *A. dahurica* Qi, *A. dahurica* Chongqing, *A. dahurica* Xiang, *A. dahurica* Taiwan, *A. dahurica* Xing'an, and so on. Currently, *A. dahurica* is used medicinally in four major medicinal varieties: *A. dahurica* Chuan, *A. dahurica* Hang, *A. dahurica* Yu, and *A. dahurica* Qi (Elith et al., 2006). The main medicinal components in *A. dahurica* herb are coumarins, including epoprostenol, epoprostenol, and oxidized prostenol (Li, 2012). It has long been used as an herbal medicine for the treatment of colds, fever-induced headaches, and pain caused by wounds (Chen and Yang, 2018). Additionally, it has a wide range of uses in food, nutraceuticals, specices, and cosmetology (Wang et al., 2020; Yang et al., 2020).

There have been many studies on the chemical composition and pharmacological effects of *A. dahurica* (Zhang et al., 2017; Li et al., 2019; Tang et al., 2021; Liu et al., 2022; Xie et al., 2022; Wang et al., 2023; Zhou et al., 2023), but reports on its distribution, the prediction and change of its habitable zone under future climate change have not yet been seen. Therefore, in order to scientifically elucidate the distribution of *A. dahurica* and its response to future climate change, this study used the optimized MaxEnt model and ArcGIS software to simulate and predict the potential distribution of the current (1970-2020), the future 2050s (2041-2060) and 2090s (2081-2100), and synthesize the contribution rate of the environmental factor variables and the replacement of the important values. Jackknife test was used to determine the appropriate intervals for the environmental factor variables, and to quantify the potential geographic distribution area and area of *A. dahurica* under threat in the future. This study aimed to: (i) predict the distribution pattern of potentially suitable areas for *A. dahurica* under current climatic conditions and classify them into different suitability classes; (ii) analyze the relationship between *A. dahurica* and the area of distribution in relation to main environmental factors; and (iii) predict the trend of variation in the centroid of *A. dahurica* within its habitat under future climate scenarios in China.

## 2 Materials and methods

### 2.1 Species distribution data of *A. dahurica*

**Figure 1:**
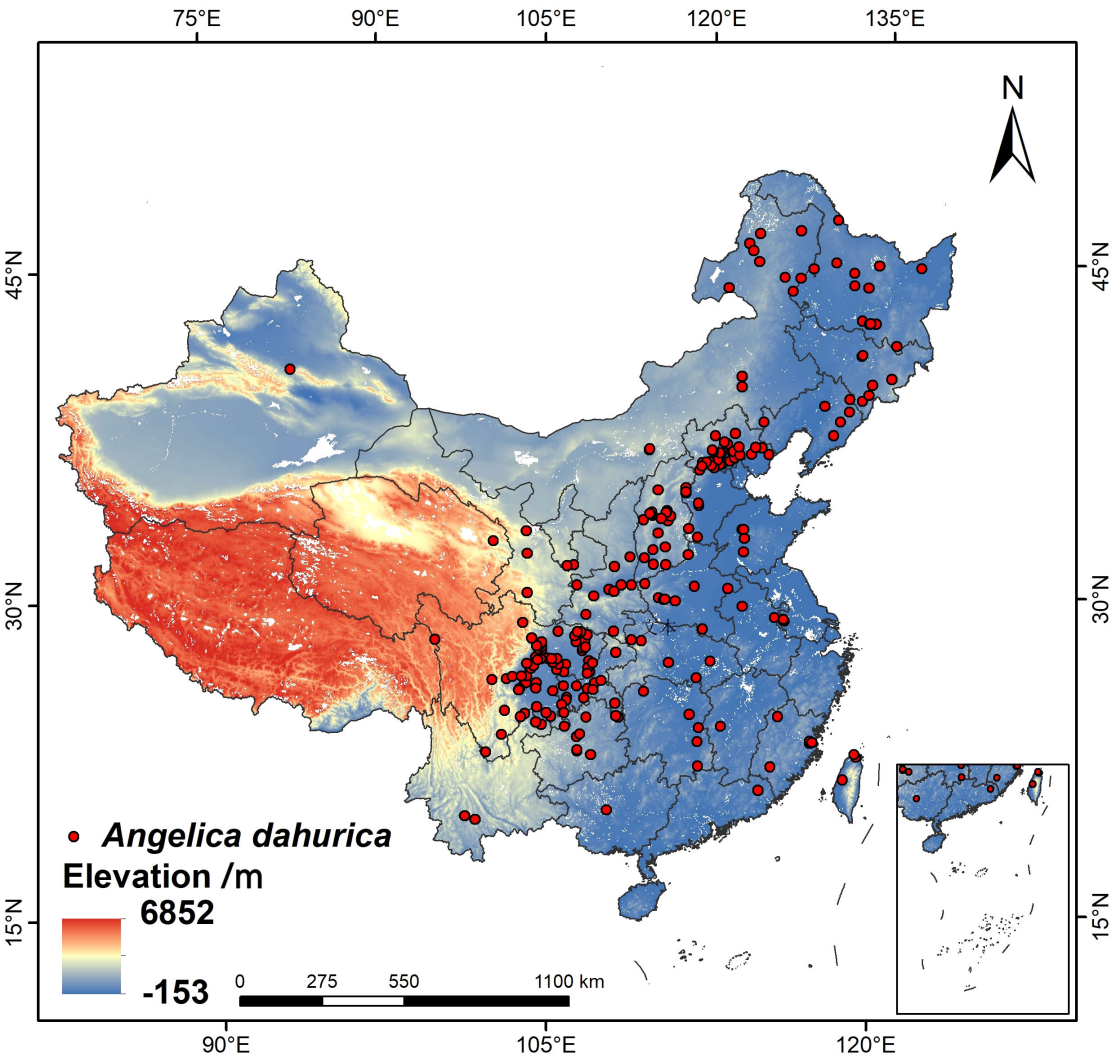
Distribution location of *A. dahurica* sampling sites.

The distribution data of *A. dahurica* in this study were obtained from the Global Biodiversity Information Facility (GBIF), the Chinese Virtual Herbarium (CVH). Only distribution data with clear latitude and longitude coordinates were retained in the data collection process, and a total of 530 distribution points were collected. After deleting the duplicated and erroneous sample points, 234 distribution record points were finally obtained, and the effective distribution locations were shown in **Figure 1**.

### 2.2 Sources and processing of environmental data

The environmental data used in this study came from the Worldclim version 2.1 (http://www.worldclim.org) environmental database, and the main environmental factors selected to affect the distribution of *A. dahurica* included 19 bioclimatic variables (bioclim) current (1970-2000), 2050s (2041-2060), and 2090s (2081-2100) as detailed in **Table 1**. Fourteen soil factors (percent gravel volume, percent gravel, percent chalk, percent clayey grains, soil texture type, soil density, percent organic carbon, pH, cation exchange of clayey constituents, cation exchange, salt base saturation, sum of exchangeable salt bases, percent exchangeable sodium salts, and electrical conductivity) were used in the 0-30 cm surface soil layer. Soil factor and topographic factor data were obtained from the Food and Agriculture Organization of the United Nations (FAO) World Soil Database (HWSD) (http://www.fao.org/faostat/en/#data). Map data were obtained from the Ministry of Natural Resources (http://www.mnr.gov.cn/), and all layer data was converted to GCS-WGS-1984 projection format. The obtained data were then masked to obtain Chinese data using the Chinese map in ArcGIS 10.4 software. Finally, all environmental data were converted to *.asc format. All environmental variables were processed through a series of treatments, and the spatial resolution of each factor was set to 2.5min (about 5 km). Three scenarios (SSP126, SSP245, and SSP585) from the BCC_CSM Future Climate System Scenarios Model (FCSM) were applied in this study, which represents the low, medium, and high GHG emission scenarios, respectively. Since excessive covariance between environmental variables can cause overfitting of the model and lead to a reduction in the transferability of the model, the study screened the variables based on Pearson’s correlation coefficient and variance inflation factor (VIF) to reduce the covariance between the variables. In this study, the R language correlation program package was utilized to identify factors with a correlation below 0.8 (Dormann et al., 2013; Merow et al., 2013). Subsequently, factors with a VIF value less than 10 were chosen. The R language correlation code was then employed to conduct a Pearson correlation test, retaining factors with a correlation coefficient below 0.8. Additionally, for factors with a correlation coefficient exceeding 0.8, only those with greater ecological significance were retained (Wang et al., 2019). A VIF value between 10 and 100 indicates multicollinearity among factors, while a VIF above 100 signifies serious interfactor multicollinearity.

**Table 1:** 19 environmental factor used in MaxEnt modeling.

Variable code	Environmental factor
Bio1	Annual Mean Temperature (°)
Bio2	Mean Diurnal Range (°)
Bio3	Isothermality (Bio2/Bio7) (×100)
Bio4	Temperature seasonality (standard deviation×100)
Bio5	Max Temperature of Warmest Month (°)
Bio6	Min Temperature of Coldest Month (°)
Bio7	Temperature Annual Range (Bio5-Bio6) (°)
Bio8	Mean Temperature of Wettest Quarter (°)
Bio9	Mean Temperature of Driest Quarter (°)
Bio10	Mean Temperature of Warmest Quarter (°)
Bio11	Mean Temperature of Coldest Quarter (°)
Bio12	Annual Precipitation (mm)
Bio13	Precipitation of Wettest Month (mm)
Bio14	Precipitation of Driest Month (mm)
Bio15	Precipitation Seasonality (mm)
Bio16	Precipitation of Wettest Quarter (mm)
Bio17	Precipitation of Driest Quarter (mm)
Bio18	Precipitation of Warmest Quarter (mm)
Bio19	Precipitation of Coldest Quarter (mm)

Six climatic factors (Annual Mean Temperature, Max Temperature of Warmest Month, Min Temperature of Coldest Month, Annual Precipitationl, Precipitation of Driest Month, Precipitation of Wettest Quarter), one topographic factor (elevation), and eleven soil factors (percentage of gravel by volume of surface soil, percentage of chalk, percentage of clayey grains, percentage of organic carbon, pH, cation exchange, saturation of the salt base, sum of exchanged salts, percentage of exchanged sodium salts, electrical conductivity, and cation exchange for clayey grains constituents), totaling eighteen environmental factors, were ultimately selected.

### 2.3 Species distribution modeling

The study used MaxEnt software for model construction. The organized species point information of *A. dahurica* was saved in CSV data format and imported into the software together with environmental variables, and then the output type was set as Logistic, 25% of the species distribution data was selected as the test set, 75% as the training data set, and all other parameters were default values. The model factor contributions were analyzed using the Jackknife measure (Pearson et al., 2006), and then analyzed by the ROC curve. The horizontal and vertical coordinates of the ROC curve represent, respectively, the rate of actual absence but predicted presence and the rate of actual presence and predicted presence, and the area enclosed by them is called the AUC. Its magnitude is used as a measure of the accuracy of the model prediction. The AUC takes values ranging from 0 to 1, with larger values indicating better prediction results, 0.9 to 1.0 indicates very good results, 0.8 to 0.9 indicates good results, 0.7 to 0.8 indicates fair results, 0.6 to 0.7 indicates poor results and 0.5 to 0.6 indicates failure of the results. Ture skill statistic (TSS) was also used to assess the net predictive success of the model and the accuracy of the model predictions.

### 2.4 Optimization of the model

In order to avoid the overfitting phenomenon, the study selects the optimal parameters by testing the AIC information criterion correction (OuYang, et al.) of Maxnent’s model with different feature combinations (FC) and regularization multiplier (RM) conditions. Referring to Robert Muscarella’s latest optimization method, the Checkerboard2 method was used to divide the study area into 4 bins, a masked geostructuring method that better adjusts the level of model regularization. The MaxEnt model regularization level contains 2 parameters, modulation multiplicity (RM) and feature combination (FC), which are optimized by calling the ENMeval (Muscarella et al., 2014) packet in R. The MaxEnt model provides five types of features, namely linear features (L), quadratic features (Q), fragmentation features (H), product features (P) and thresholding features (T).In this study, the default parameters of MaxEnt software are RM=1, FC=LQHPT; in order to optimize the MaxEnt model, RM is set to 0.5-4, increasing by 0.5 each time, for a total of 8 modulation octaves, while 6 combinations containing 1 or more features are used:L; LQ; H; LQH; LQHP; LQHPT; and 48 kinds of calculations are made based on the permutation and combination of parameter combinations. The ENMeval data package examines the 48 parameter combinations mentioned above to test the complexity of the model based on the delta. The AICc value and the 10% test omission rate are both indicators of the accuracy of model predictions. The lower these values are, the more accurate the model predictions will be.

### 2.5 Data processing

ArcGis10.4 software was used to process the division and visualization of the suitability of *A. dahurica*. Based on the suitability threshold of *A. dahurica* predicted by the MaxEnt model, the habitat suitability index of *A. dahurica* was classified using the natural breakpoint method. The habitat suitability of *A. dahurica* was categorized into unsuitable area (0∼0.1), low suitable area (0.1∼0.29), middle suitable area (0.29∼0.5), and high suitable area (0.5∼1). We compared the differences in the suitable zones of *A. dahurica* in different periods, so as to obtain the spatial distribution pattern of *A. dahurica* under future climate change scenarios. Using the SDMTool data package in R, the center of mass positions of the fitness zone domains of *A. dahurica* were calculated for the current and the future three climate scenarios, and the range shift direction of the spatial distribution of the fitness zone of *A. dahurica* was reflected by the change of the center of mass positions; the center-of-mass range shift distances of *A. dahurica* under different climate scenarios were counted using the geosphere (https://cran.r-project.org/package=geosphere) data package in R language.

## 3 Results and analysis

### 3.1 Model optimization and accuracy evaluation

**Figure 2:**
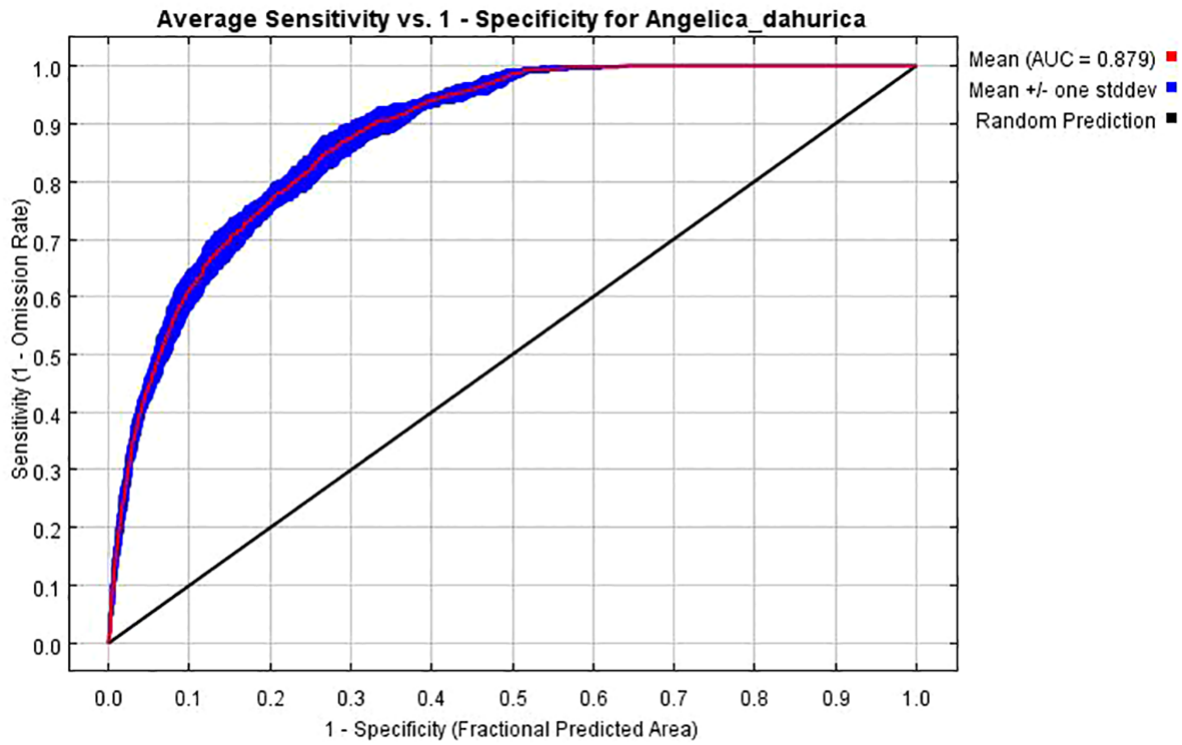
Certification of ROC curve of MaxEnt model on the prediction result of *A. dahurica*.

Based on 234 A*. dahurica* distribution points and 18 environmental variables layers, and the AICc information criterion, the potential distribution area of *A. dahurica* was simulated and predicted using the MaxEnt model. With MaxEnt default parameter settings, the RM=1, the FC=LQHPT, and the delta.AICc=8.09353. We optimized the model to obtain results when delta.AICc = 0, RM = 1.5 and FC = LQHPT, when the model is optimal and the 10% training omission value is lower than that of the model with default parameters (**Table 2**). For this reason, the modulation multiplier RM=1.5 and FC=LQHPT were selected as the final parameters of the model, and the AUC value of the simulated training under this parameter was 0.879 (**Figure 2**), which indicates that the prediction results were accurate. The TSS value was 0.62. Therefore, these assessment methods showed that the accuracy of using the model to predict the distribution of potentially suitable habitats for *A. dahurica* was relatively high.

**Table 2:** Evaluation results of MaxEnt model with different parameter settings.

Model evaluation	Feature combination	Regularization multiplier	Value of delta akaike information criterion corrected	10% training omissiion rate
Default	LQHPT	1	8.09	0.17161
Optimized	LQHPT	1.5	0	0.15875

### 3.2 Potential geographic distribution of *A. dahurica* in China in the current period

**Figure 3:**
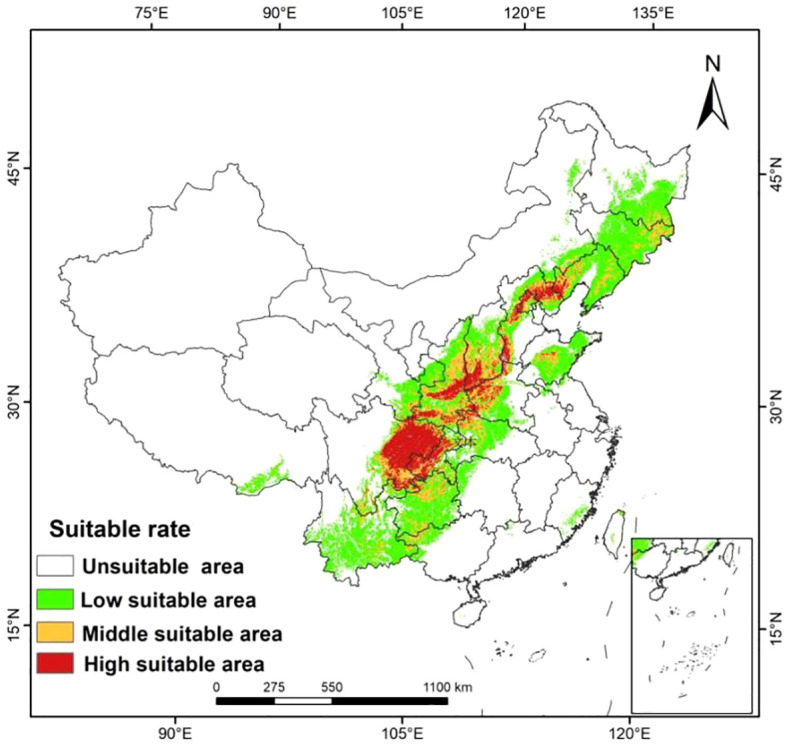
The distribution of suitable growing areas of *A. dahurica* in China predicted by the MaxEnt model.

The Maxent model was used to simulate the distribution map of suitable areas for *A. dahurica* in the current period (**Figure 3**). The high suitable area and middle suitable area of *A. dahurica* in the current period were 16,159 km2 and 24,849 km^2^, respectively. These areas were mainly located in Yunnan, Guangxi, Guizhou, Hunan, Sichuan, Chongqing, Henan, Hubei, Gansu, Shanxi, Shaanxi, Hebei, Liaoning, Jilin, Heilongjiang, and other regions. The high suitable areas were concentrated in eastern and southeastern Sichuan, Chongqing, central Shaanxi, southwestern Shanxi, central Hebei, and Liaoning. Additionally, there were small areas of optimal habitat in Shandong and Guizhou.

### 3.3 Distribution and geographic variation of *A. dahurica*’s predicted aptitude zones

**Figure 4:**
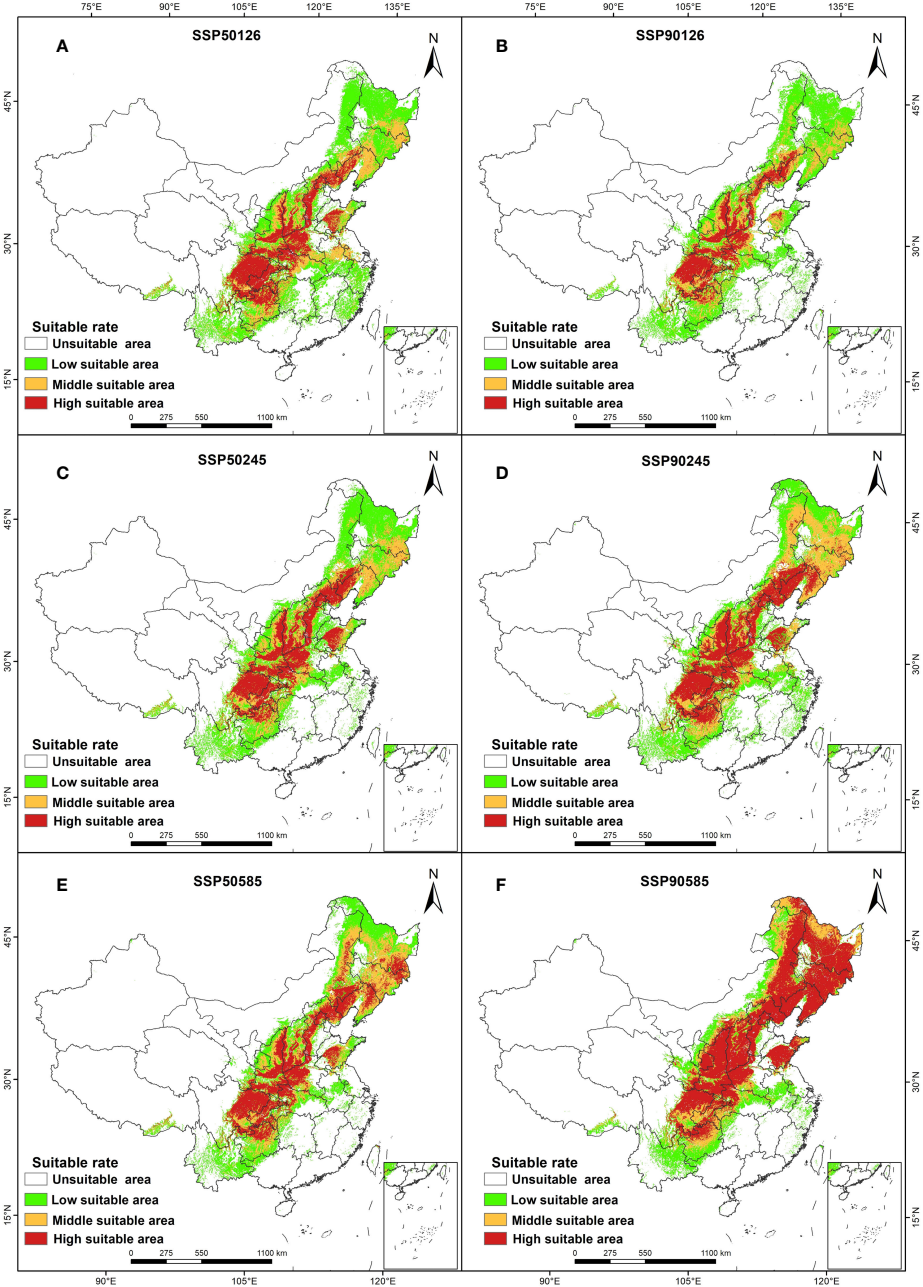
Distribution of suitable areas for *A. dahurica* in China in ssp126 **(A, B)**, ssp245 **(C, D)**, ssp585 **(E, F)** scenarios in the 2050s **(A, C, E)** and 2090s **(B, D, F)**.

**Figure 5:**
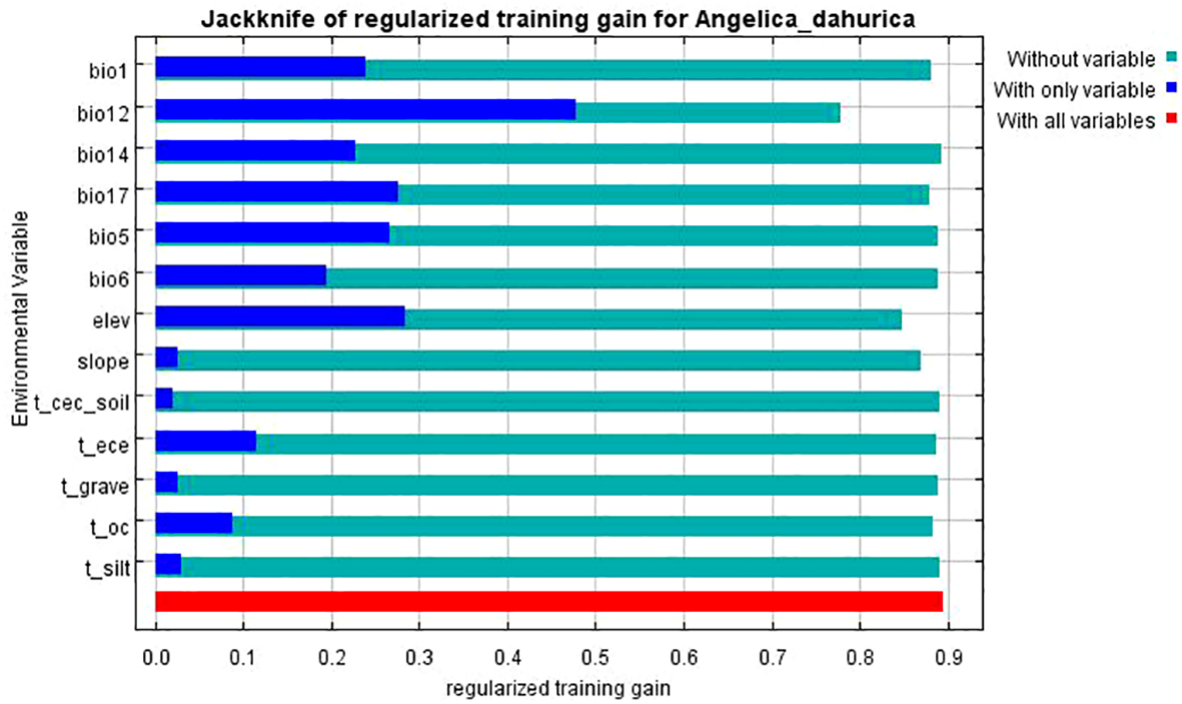
Variation of geographic distribution of the center of mass of *A. dahurica* aptitude zone under different climate change scenarios.

Comparing the predicted suitable areas of *A. dahurica* in the 1950s and 1970s, we found that the response of *A. dahurica* to climate change varied, with different trends (**Figures 4**, **5**). Under the SSP245 scenario, the area of the high suitable area for *A. dahurica* in the 2090s showed the smallest change, with an increase of 12,903 km^2^ compared to the current situation, while under the SSP585 scenario, the area of the high suitable area for *A. dahurica* showed the largest change, with an increase of 36,116 km^2^ in the high suitable area by the 2050s. The current total suitable area is 109,898 km^2^, accounting for 20.6% of the total land area (**Table 3**); the suitable area of *A. dahurica* showed an expanding trend under all three climate scenarios and was most sensitive to climate change under the SSP585 scenario.

**Table 3:** Area of suitable areas for *A. dahurica* under different future climate scenarios.

scenarios	Unsuitable area	Low suitable area	Middle suitable area	High suitable area	Total suitable area
Current	424,536	68,890	24,849	16,159	109,898 (20.6%)
SSP126 2050S	369,528	85,674	41,622	37,610	164,906 (30.9%)
SSP126 2090S	376,686	78,986	38,549	40,213	157,748 (29.5%)
SSP245 2050S	380,682	60,614	45,933	47,205	153,752 (28.8%)
SSP245 2090S	389,906	79,170	36,296	29,062	144,528 (27.0%)
SSP585 2050S	359,408	69,092	53,659	52,275	175026 (32.7%)
SSP585 2090S	350,797	43,784	37,682	102,171	183,637 (34.4%)

**Figure 6:**
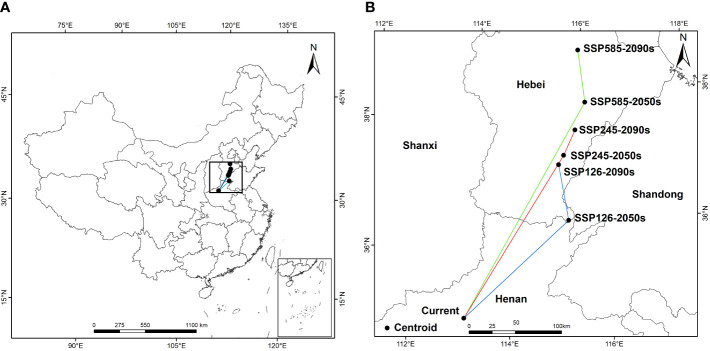
Geographical distribution changes of the centroid of the suitable area of *A. dahurica* under different climate scenarios [**(B)** is an enlargement of the part of **(A)**].

In terms of spatial pattern, there were differences in the shift change of the suitable area of *A. dahurica* under different climate scenarios, but the overall range shift trend was more consistent, with the overall trend range shifting to the north (**Figure 6**). In the current period, the centroid of *A. dahurica* suitable area is in Zhengzhou, Henan Province. When the climate scenario is SSP126-2090s, the centroids of *A. dahurica* fertile zone shifted to the northeast, and at this time, the centroid of *A. dahurica* fertile zone is located in Shijiazhuang, Hebei Province, with a range shift distance of 250,359 m. When the climate scenario is SSP585-2090s, the centroid of *A. dahurica* fertile zone shift northward, and it is moved to Baoding, Hebei Province, with a shift distance of 250,275 m. In the future, under the climate change scenario, global warming and humidification will cause the centroid of the fertile zone in China to range shift northward, and there is a tendency for further northward expansion of range shift. Under the future climate change scenario, global warming and humidification will cause the center of mass of *A. dahurica* fertile zone in China to range shift to the north, and the range shift position will have a tendency to further expand to the north.

### 3.4 Primary Environment Variables

**Figure 7:**
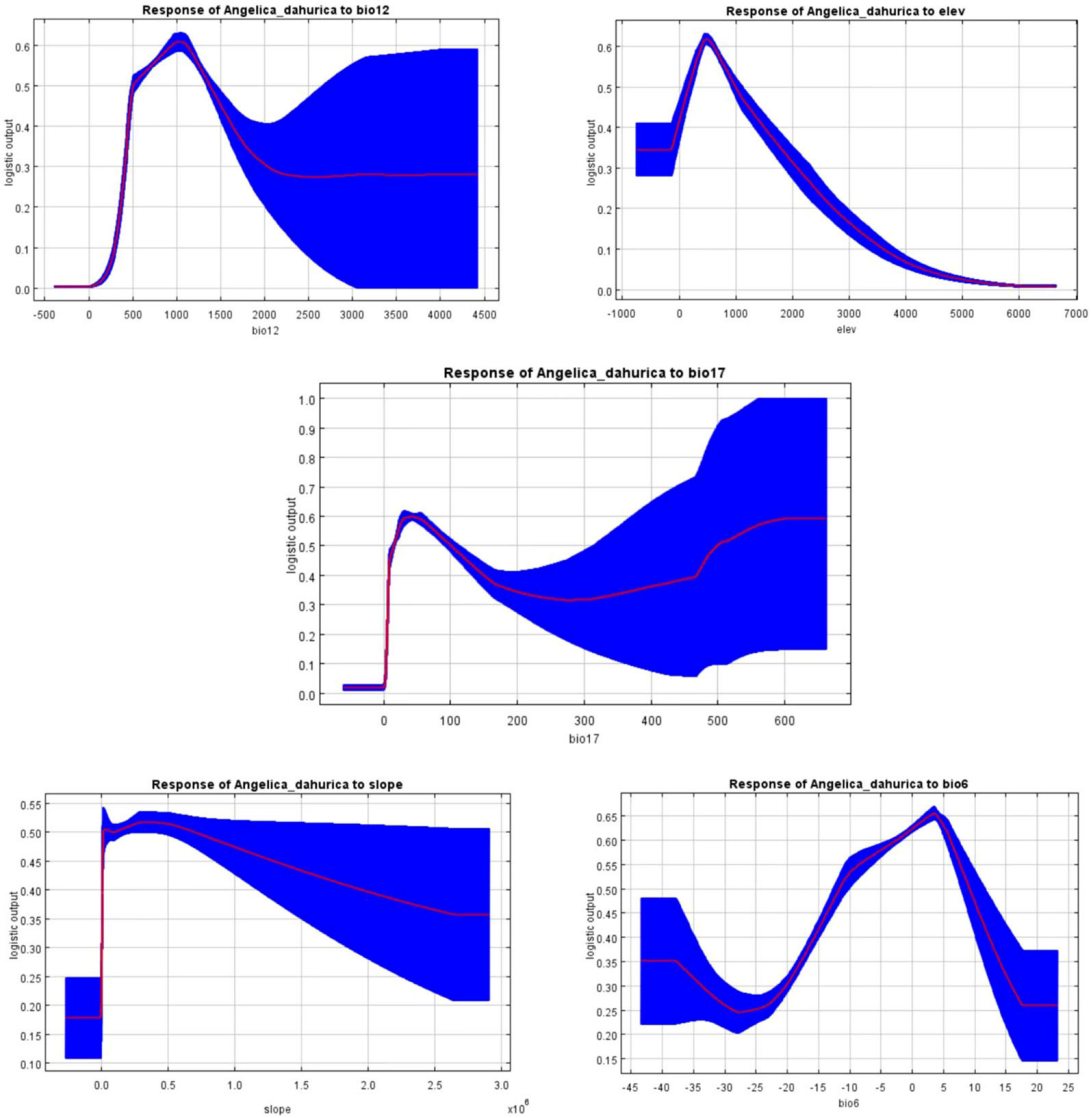
Response curves for dominant environmental factors.

Based on the results of the Jackknife analysis (**Figure 7**), the contribution rate of each environmental variable to the model construction was calculated, and the analysis results are shown in **Table 4**. The factors that contributed more than 4% to the potential geographic distribution of *A. dahurica* were annual rainfall (51.4%), elevation (23.3%), slope (4.2%), rainfall in the driest season (6.3%), and minimum temperature in the coldest month (4.4%), and the total contribution rate of these five environmental factors was as high as 89.6%, and the importance value of these five environmental factors was 84% at the same time, the total contribution of these five environmental factors was 89.6%, while the importance value of these five environmental factors was 84%.

**Table 4:** Contributions and Significant Values of Environmental Factors.

Varible	Percent contribution	Permutation importance
Bio12	51.4	50.7
Elevation	23.3	18.2
Bio17	6.3	9.5
Bio6	4.4	2.2
Slope	4.2	3.4

**Figure 8:**
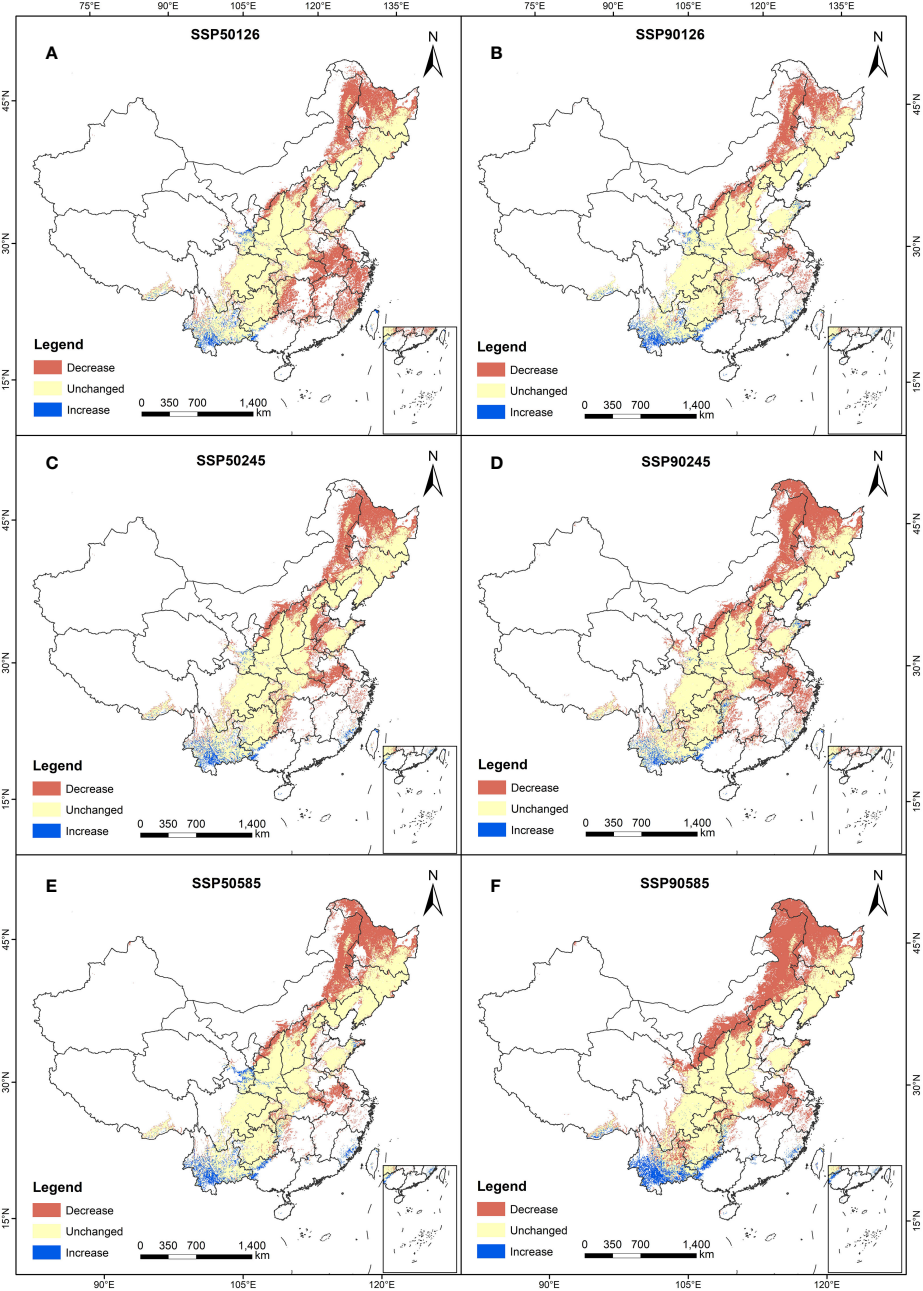
Spatial changes in *A. dahurica*. in China in ssp126 **(A, B)**, ssp245 **(C, D)**, ssp585 **(E, F)** scenarios in the 2050s **(A, C, E)** and 2090s **(B,D, F)**.

The main environmental variables were selected to draw the response curves of environmental variables, which are shown in **Figure 8**. From **Figure 8**, it can be seen that: annual precipitation has the greatest influence on the geographic distribution of *A. dahurica*, and the probability of the existence of *A. dahurica* is extremely small when the rainfall of the current year is < 0 mm; When the annual precipitation is 0~1000 mm, the existence probability increases rapidly, and it is the highest at about 1043mm, with the survival probability reaching 0.61. After 1000mm, it shows a decreasing trend and tends to be stabilized at 2500 ~ 4418 mm, with the survival probability of 0.27, indicated that *A. dahurica* has a high sensitivity to precipitation. There is an increasing trend from 0 to 491 m above elev, reaching a maximum at 491 m, with the survival probability of 0.62; after that the survival probability decreases sharply with increasing altitude; The probability of the presence of *A. dahurica* was greatest when the precipitation of driest quarter was 43 mm, and the survival probability of the presence of *A. dahurica* was low at a slope of < 0. It increased rapidly from 0 to 0.5*10^6^, and reached a maximum at 0.3*10^6^, and then decreased gradually. The min temperature of coldest month shows an upward and then downward trend as the temperature rises, and shows an upward trend and increases to a maximum and then decreases at 3°, suggested that hot weather can limit the expansion of *A. dahurica*, which could explain the current phenomenon of low fitness or unfitness of *A. dahurica* in some hot areas. The above results correspond to the growth habit of *A. dahurica*.

The relationship between major environmental factors and potential distribution areas is shown in **Table 5**. Under the SSP126 climate scenario, the 2050s suitability of *A. dahurica* is 0.07 higher than the current, while the 2090s suitability is 0.04 higher than the current; under the SSP245 climate scenario, the habitat suitability of *A. dahurica* in the 2050s and 2090s was 0.06 and 0.09 higher than the current one respectively; under the SSP585 climate scenario, the habitat suitability of *A. dahurica* still showed an increasing trend, with the habitat suitability of *A. dahurica* in the 2050s being 0.60, which was 13.2% higher than the current one, and that of *A. dahurica* in the 2090s being 0.65, which was 22.64% higher than the current one 22.64%. Annual rainfall at 235 A*. dahurica* distribution sites showed the same increasing trend as the change in habitat suitability of *A. dahurica*. In the 2050s, annual rainfall increased by 1.1%, 11.8% and 5.9% for the SSP126, SSP245 and SSP585 scenarios, respectively; in the 2090s compared to the 2050s, the annual rainfall increased by 7.8%, 0.71%, and 8.9% for scenarios SSP126, SSP245, and SSP585, respectively. 235 A*. dahurica* distribution sites showed an incremental trend in the driest season rainfall under scenario SSP126. However, there is a decreasing trend in both the SSP245 and SSP585 scenarios; the driest season rainfall is lower than the current in the 2050s and 2090s in both the SSP245 and SSP585 scenarios, and the driest season rainfall is higher than the current in the 2050s only in the SSP126 scenario. In 2050s, the total fitness zone of *A. dahurica* increased by 50.05%, 39.90% and 59.26% in SSP126, SSP245 and SSP585 scenarios, respectively; in the 2090s the total fitness zone domain showed a decreasing trend in SSP126 and SSP245 scenarios compared to 2050s, and showed an increasing trend in SSP585, with an increase of 4.92%.

**Table 5:** Analysis of the results of the main environmental factors.

Variable	current	SSP126		SSP245		SSP585	
		2050s	2090s	2050s	2090s	2050s	2090s
Bio12	862.21	871.84	939.41	924.41	931.03	913.2	994.34
Bio17	42.56	39.99	42.90	41.18	40.18	42.04	40.28
Bio6	-7.44	-5.24	-4.75	-4.06	-2.71	-3.94	-2.18
Elevation	773.98	773.98	773.98	773.98	773.98	773.98	773.98
Slope	245,018.6	245,018.6	245,018.6	245,018.6	245,018.6	245,018.6	245,018.6
Suitability of Species habitat	0.53	0.60	0.57	0.59	0.62	0.60	0.65

## 4 Discussion

### 4.1 Optimization model for the prediction of *A. dahurica’s* current habitable zone

The development of applied ecology provides a powerful model for the prediction of the potential distribution of species, and the algorithms of many models have been computer-programmed and formed into software tools, among which the MaxEnt is the model with the best accuracy of prediction of the distribution of species proved by many researches at present. MaxEnt-based simulation of species distribution is a research hotspot in related disciplines such as conservation ecology and biogeography. In this study, the optimized MaxEnt model was used to predict the habitat area of *A. dahurica* and the model accuracy was evaluated using ROC curves. The results showed that the AUC value was 0.879, which indicated that the model had a good prediction effect and the reliability of the model prediction was high. In general, with a large sample capacity, the model prediction accuracy is high, and the MaxEnt model can be successfully predicted with a sample size of ≥5, and the prediction results are more accurate (Pearson et al., 2006).

*A. dahurica* is a plant with significant medicinal value. Utilizing GIS technology and the R language, 234 geographical survey data points and 34 environmental factors of *A. dahurica* were analyzed. The default parameters of the Maxent model were adjusted using the ENMeval package of R language to create an optimized Maxent model for analyzing the plant's geographical distribution in its current habitat.

Pearson correlation analysis and variance inflation factor (VIF) were used to identify the necessary factors for modeling. The Jackknife method was employed to determine the dominant environmental factors within the suitable habitat zone. Furthermore, the future changes in *A. dahurica's* geographic distribution under various climatic scenarios were explored based on the 5th climate model released by the IPCC. The impacts of climate change on *A. dahurica* growing areas in China are predicted to provide the scientific basis for the protection, development and utilization of *A. dahurica* resources under the background of climate change.

### 4.2 Constraints on the geographical distribution of *A. dahurica* by environmental variables

In terms of environmental factors, the factors that have the greatest influence on the distribution of *A. dahurica* in China are elevation and precipitation: bio6 (Min Temperature of Coldest Month), bio12 (Annual Precipitation), bio17 (Precipitation of Driest Quarter), elevation, and slope. This is closely related to the growth characteristics of *A. dahurica*, and warm and humid climate is suitable for planting in deep, loose and fertile sandy loam soil. The prediction results concluded that *A. dahurica* in China is suitable for Yunnan, Guizhou, Guangxi, Sichuan, Chongqing, Hunan, Hubei, Fujian, Jiangxi, Chongqing, Shanxi, Shaanxi, Gansu, Shandong, Hebei, Liaoning, Jilin, Heilongjiang and other provinces and municipalities in local areas. The Maxent model predicted that under the background of climate change *A. dahurica* germplasm resources was of great significance and that these areas were the preferred places for the introduction of *A. dahurica* cultivation.

### 4.3 Impact of climate warming on the geographical distribution of *A. dahurica*

Under future climate change scenarios, the distribution of suitable areas for *A. dahurica* will change to varying degrees, with an increase in suitable areas projected by the 2050s and 2090s. The potential geographic distribution of *A. dahurica* is influenced by climate, soil, and terrain factors, with climate having the greatest impact on the range shift. The model has been optimized to accurately predict the potential geographical distribution of *A. dahurica*. Future climate change resulting from global warming will alter the distribution pattern of *A. dahurica*. Results from Maxent simulations indicate a tendency for the total area of the suitable zone to increase, with expansion primarily occurring in middle and high latitudes while decreasing in low latitudes. The centers of the suitable zone for *A. dahurica* in the 2050s and 2090s are projected to shift towards higher latitudes, with the overall range shifting towards the North China Plain and the Northeast Plain. Currently, 175 points fall within protected areas. By 2050, it is projected that 197, 196, and 193 points will be within protected areas under the SSP126, SSP245, and SSP585 scenarios, respectively. By 2090, 189, 203, and 204 points are projected to be within protected areas under the three scenarios, respectively.

### 4.4 Potential limitations of the Maxent model

The Maxent model is data-dependent, and in this study we only collected data from distribution sites in China with the aim of predicting the potential distribution area of *A. dahurica* in China, so the response curves of the environmental factors could not fully reflect the response of *A. dahurica* to the environment, especially in its global distribution area. Secondly, this study only predicted the effects of climate, soil and topography on *A. dahurica*, and did not take into account the effects of species interactions, human activities and other bio-ecological factors, so the predicted potential suitability zones will deviate from the actual suitability zones.

## 5 Conclusion

In this study, the default parameters of the model were optimized, resulting in the optimal model feature combination of FC=LQHPT and a regulation multiplicity of 1.5. The average value of the area under the ROC curve (AUC) was 0.879, indicating that the prediction accuracy of the Maxent model for the potential habitable zone of *A. dahurica* was high.The results revealed that the distribution of *A. dahurica* was significantly influenced by five environmental variables: bio6 (Min Temperature of Coldest Month), bio12 (Annual Precipitation), bio17 (Precipitation of Driest Quarter), elev (Altitude), and slope (Slope variability). These factors accounted for 89.6% of the cumulative contribution to the distribution of *A. dahurica*. *A. dahurica* showed that high sensitivity to precipitation with hot weather potentially limited its expansion. Based on the study, *A. dahurica* was most suitable for regions in Shanxi, Shaanxi, Gansu, Shandong, Hebei, Liaoning, Jilin, Heilongjiang, and most provinces and cities south of the Yangtze River in our country. The suitable area for *A. dahurica* was increasing overall, with expansion mostly occurred in middle and high latitudes while reductions were observed in low latitudes. Considered three future climate change scenarios, it was found that the centers of mass for *A. dahurica*'s suitable areas in the 2050s and 2090s were shifted towards higher latitudes, with the entire suitable area moving towards the North China Plain and Northeast Plain. Thus, it was of great significance to use the Maxent model to predict the development and utilization of *A. dahurica* germplasm resources in the face of climate change.

## Data availability statement

The original contributions presented in the study are included in the article/supplementary material. Further inquiries can be directed to the corresponding author/s.

## Author contributions

F-GZ: Data curation, Formal Analysis, Investigation, Writing – original draft. FL: Conceptualization, Methodology, Software, Writing – review & editing. KW: Investigation, Validation, Writing – review & editing. LX: Investigation, Validation, Writing – review & editing. GZ: Resources, Supervision, Writing – review & editing. YW: Funding acquisition, Supervision, Validation, Writing – review & editing.
